# The emergence of the vertical birth in Ecuador: an analysis of agenda setting and policy windows for intercultural health

**DOI:** 10.1093/heapol/czv118

**Published:** 2016-01-11

**Authors:** Ana Llamas, Susannah Mayhew

**Affiliations:** London School of Hygiene and Tropical Medicine 15-17 Tavistock place, London, WC1H 9SH.

**Keywords:** Agenda setting, Ecuador, intercultural health, indigenous health, maternal health, policy

## Abstract

Maternal mortality continues to claim the lives of thousands of women in Latin America despite the availability of effective treatments to avert maternal death. In the past, efforts to acknowledge cultural diversity in birth practices had not been clearly integrated into policy. However, in Otavalo (Ecuador) a local hospital pioneered the implementation of the ‘Vertical Birth’—a practical manifestation of an intercultural health policy aimed at increasing indigenous women’s access to maternity care. Drawing on agenda-setting theory, this qualitative research explores how the vertical birth practice made it onto the local policy agenda and the processes that allowed actors to seize a window of opportunity allowing the vertical birth practice to emerge. Our results show that the processes that brought about the vertical birth practice took place over a prolonged period of time and resulted from the interplay between various factors. Firstly, a maternal health policy community involving indigenous actors played a key role in identifying maternal mortality as a policy problem, defining its causes and framing it as an indigenous rights issue. Secondly, previous initiatives to address maternal mortality provided a wealth of experience that gave these actors the knowledge and experience to formulate a feasible policy solution and consolidate support from powerful actors. Thirdly, the election of a new government that had incorporated the demands of the indigenous movement opened up a window of opportunity to push intercultural health policies such as the vertical birth. We conclude that the socioeconomic and political changes at both national and local level allowed the meaningful participation of indigenous actors that made a critical contribution to the emergence of the vertical birth practice. These findings can help us advance our knowledge of strategies to set the agenda for intercultural maternal health policy and inform future policy in similar settings. Our results also show that Kingdon’s model was useful in explaining how the VB practice emerged but also that it needs modifications when applied to low and middle income countries.

Key Messages

- Kingdon’s model was useful in explaining how this policy emerged but it needs modifications when applied to low- and middle-income countries.
- The presence of a strong indigenous movement and changes at the socioeconomic and political level were critical factors in the emergence of the vertical birth policy.
- Intercultural health policies addressing maternal health can emerge when indigenous actors engage meaningfully in political processes to integrate their values into policy.

## Introduction

As we move into a post-Millennium Development Goals (MDGs) era, maternal mortality remains unacceptably high (UN 2014) despite the availability of effective treatments to avert maternal deaths (Campbell and Graham 2006). Across the world, indigenous people bear the burden of ill health (Casas *et al.* 2001; Montenegro and Stephens 2006). Indigenous health is characterized by stark inequities^1^ between the indigenous and non-indigenous population that are the result of socioeconomic factors combined with historical and culturally specific factors (King *et al.* 2009). Ecuador is no exception. Indigenous people are amongst the most disadvantaged in society (Hall and Patrinos 2010) and, although estimating indigenous maternal mortality is difficult because data are not disaggregated by ethnicity, process indicators show sharp contrasts between indigenous and non-indigenous women. For example, in Ecuador only 30% of indigenous women had a skilled attendant at birth compared to 80% of non-indigenous women (Endemain 2004).

It is now recognized that to reduce maternal mortality amongst indigenous women health services need to be more responsive to their social and cultural context (Medina Ibanez 2006; Kayongo *et al.* 2006; Goicolea *et al.* 2008; Ortega 2010). Certainly, cultural barriers prevent indigenous women from accessing health services, even when financial and geographical barriers are lowered (Conejo 1998; Camacho *et al.* 2006; PAHO 2008). ‘Intercultural health’ is a concept that has emerged over the past few years to encompass the idea of strategies to articulate both traditional and western medicine under the premise of mutual respect and recognition for both medical systems (Mignone *et al.* 2007). In other words, it is a meeting of two medical traditions often seen as culturally opposed (thus ‘inter-cultural’).

In this case study, intercultural health policy was manifested (practically implemented) at the local level through the vertical birth (VB) practice. Across Latin America, similar intercultural health initiatives have spawned to facilitate indigenous people’s access to care (Gabrysch *et al.* 2009; ; Guerra-Reyes 2009; Tucker *et al.* 2013; van Dijk *et al.* 2013). There are examples of intercultural health policies (i.e. strategies undertaken to achieve specific healthcare goal) that have been very successful in increasing indigenous women’s access to skilled birth attendants. In Peru, for instance, a similar practice to the VB described in this article achieved an institutional delivery increase from 6 to 83% (Gabrysch *et al.* 2009).

Given the potential for intercultural health policies to improve maternal health outcomes among indigenous women, it is important to understand how these policies are developed and championed. Otavalo is an interesting case study because this is not the area of the country with the highest maternal mortality rate (MMR) (INEC 2012) yet it has seen sustained attention to reproductive health and pioneered the VB practice which was upheld as the primary example of how the intercultural health policy could be implemented. In this sense the VB is ‘policy with a small p’ as envisaged by Meyerson *et al.* (2014) while the intercultural health policy is the ‘policy with a big P’ (the policy sanctioned by government). To our knowledge, this is the first study that aims to understand the factors influencing the emergence of a practice (or ‘policy with a small p’) that represents the implementation of a national intercultural health policy. We draw on one of the most widely used models of agenda setting (Kingdon 2003) to qualitatively explore the emergence of the VB in Otavalo. According to Kingdon (2003), policy emerges from the convergence of three different streams or processes: the problem stream, the policy stream and the politics stream. The problem stream refers to how we come to understand something as a problem that is worth considering. Indicators, focusing events and feedback from existing programs draw officials’ attention to the problem. The policy stream is the process by which proposals are developed and selected for serious consideration. Those policies that are technically feasible, acceptable, coherent with the values of the community, and capable of dealing with future constraints are more likely to be seriously considered. Finally, the politics stream relates to political events such as changes of government. These streams run independently but occasionally converge to create a policy window when policy change is likely to occur. According to Kingdon’s model, policy entrepreneurs are actors inside and outside the government who identify these policy windows and move issues onto the government agenda (Kingdon 2003). In this article, we assess the existence of these three streams and examine the factors that contributed to a window of opportunity being taken to secure the implementation of the VB practice (policy with a small p) as a manifestation of the national intercultural health policy in Otavalo, Ecuador.

## Methods

### Study setting

Otavalo is located in the Ecuadorian Andean mountains. This is one of the areas of the country with highest concentration of indigenous people (INEC 2001; MSP 2010). Otavalo and surrounding areas are considered the cradle of the indigenous movement (Lalander and Gustafsson 2001, 2008) which is one of the strongest in Latin America (Lalander 2010). In the early 1990s, the indigenous movement burst into the national political scene when they staged several popular uprisings that culminated in the toppling of two national governments (Lalander and Gustafsson 2008; Becker 2011; Bowen 2011). Since then, the indigenous movement became an influential political force in the country which achieved formal recognition of cultural and political autonomy (Bowen 2011) and influence over some policy areas such as bilingual education, rural development and institutional design (Lalander and Gustafsson 2008; Bowen 2011). The indigenous movement was also successful in local elections. In 2000, an indigenous leader won the mayoral elections for the first time in Otavalo (Lalander 2005) and remains in power to date.

The city is well known for its market of indigenous handicrafts and music which attracts large numbers of international and national tourists (Lalander 2005). This economic activity has made Otavalo’s indigenous community one of the wealthiest in Latin America (Bowen 2011). However, economic development has been uneven; wealthier indigenous people tend to live in urban areas whilst the poorer majority live in rural areas, where access to intra-partum care is more difficult. There are private providers but the Ministry of Health (MOH) is the main health service provider in Otavalo; there is a hospital with 75 beds and numerous primary health clinics in rural and urban areas. Public services are decentralized: Otavalo health area is coordinated by the provincial department of health which in turn reports to the national MOH.

### The vertical birth practice

In 2007, the public hospital in Otavalo launched the VB, an intercultural health practice seeking to facilitate indigenous women’s access to intra-partum care and thus to improve maternal and neonatal health outcomes amongst this ethnic group. Although the VB practice involved more than allowing women to deliver upright, ‘vertical birth’ is the name given to the practice by respondents, policy documents, media, etc. and we therefore retained it. In fact, the VB practice entailed building a delivery room within the hospital’s premises that resembled a traditional indigenous house, including a small space to prepare herbal remedies. The room was equipped with ropes and bars that allowed women to adopt vertical positions during delivery, which was considered a critical factor in traditional indigenous birth practices. Women were allowed to mobilize during labour, to have a relative with them and efforts were made to maintain women’s modesty by providing them with appropriate gowns and installing curtains in the dilatation room. Several workshops were carried out with hospital staff to raise awareness about intercultural health and basic use of Kichwa language (the main indigenous language) in clinical settings was taught. Health professionals were trained to deliver women in vertical positions. In addition, traditional birth attendants (TBAs) were incorporated into the labour ward. Their role was to support women and provide traditional care (e.g. herbal remedies) throughout labour in collaboration with health professionals (MSP 2010). The VB was widely hailed as a success; numerous national and international delegations visited Otavalo to see the maternity unit first-hand and it was being used as a model to replicate elsewhere and informed national guidelines on intercultural maternity care.

### Data collection

We interviewed a total of 15 respondents (8 indigenous and 7 mestizo white/mixed race people) involved in setting the agenda for the VB practice in Otavalo. We conducted semi-structured interviews from October 2009 to December 2010 with health managers working for the MOH and an NGO (*n*  =  8), policy-makers, including politicians, and civil servants (*n* = 5) and community leaders (*n* = 2) involved in indigenous health and maternal health at the local, provincial and national level. These actors were identified and recruited through informal and formal interviews and through snowballing.

Interviews were conducted in Spanish and included questions about their views on the VB practice and the context and particular factors that led to its emergence. Interviews were digitally recorded and hand written notes were taken. Verbatim transcriptions of the interviews were then prepared for analysis. As part of the research, the principal investigator (PI) conducted observation in the local hospital and two PHC clinics. The PI also attended a FGD with the main actors involved in maternal and indigenous health in Otavalo, several workshops, conferences and meetings related to indigenous maternal health, she collected relevant policy documents (e.g. national guidelines on intercultural health and maternal health, legal documents, and local hospital protocols) and health statistics and took extensive field notes.

Qualitative data were analysed manually and using Nvivo 8 package. Policy documents and field notes were used to inform interview guides, triangulate results and contextualise findings. Initially, a thematic content analysis was conducted to identify and describe the key elements of respondents’ accounts (covert and overt themes). We then looked for relationships and associations between different themes to understand the VB practice emergence and the main factors that facilitated it (Ritchie and Lewis 2003; Green and Thorogood 2009). We compared respondents’ accounts and looked for divergent cases to refine our analysis (Green and Thorogood 2009; Silverman 2010). We also drew on political and social literature to guide our analysis (Walt 1994; Kingdon 2003; Buse *et al.* 2005; Shiffman 2003; Shiffman and Smith 2007; Sabatier 2007 ). Data derived from interviews were then triangulated as a way of clarifying conflicting information, providing a fuller picture of the research problem (Ulin *et al.* 2002; Ritchie and Lewis 2003) and enhancing reflexivity (Ulin *et al.* 2002). The data excerpts selected here are used to illustrate typical findings and/or eloquent explanations of the phenomena studied (Green and Thorogood 2009). The main study received ethical approval from the London School of Hygiene & Tropical Medicine. In Ecuador, the study was approved by the University of Otavalo and other stakeholders, including the hospital director. Respondents were given information about the research and verbal or written consent was obtained prior to each interview.

This paper is structured according to Kingdon’s three streams of agenda setting. It first explores the key factors influencing the defining of indigenous maternal health as a ‘problem’ to be addressed (problem stream). The article then analyses how solutions leading up to the VB practice were developed (policy stream). Finally, the political events that facilitated the eventual emergence of the VB practice are identified (politics stream). Based on these findings, the final section the article discusses the interplay of influences on the emergence of the VB practice and offers a critique of Kingdon’s model and its utility in explaining the emergence of a ‘small p’ policy in a middle income country.

## Results

### Problem stream: indigenous rights movement help define national and local maternal rights and policy communities

In this section, we first explore how indigenous leaders came to be in positions of health policy decision-making. This is critical for understanding the framing of MMR differentials as a ‘problem’ of ethnic inequity in maternal health outcomes for indigenous vs. non-indigenous women. The development of this ‘ethnic inequities’ frame is explored in the second subsection, which also examines disagreements within the policy community. In these sections, we also draw attention to the interaction between local and national actors that helped shape the VB practice and intercultural health policy more broadly.

### Formation of an influential maternal health policy community

Core to formulating indigenous maternal health inequities as a serious health problem requiring government intervention, was the consolidation of a body of actors who championed indigenous health and rights. Interview data shed light on how indigenous health champions emerged in Otavalo who could formulate ‘the problem’. Data show that a maternal health policy community emerged that consisted of a relatively stable group of indigenous and mestizo healthcare workers (HCWs), managers and policy-makers involved in maternal and indigenous health in Otavalo. This policy community was in part the product of the particular socio-economic and political history of Otavalo.

Respondents described how over a generation, Otavalans’ success as global merchants and the availability of scholarships allowed some indigenous people to access higher education, including medical training. Some of these young indigenous professionals were committed to improve living conditions for the poorer indigenous community and, as they gradually took jobs in institutions such as the MOH, the local government and civil society organizations, they were able to shape local policy. Their ability to influence local health policy was further increased by events in the national political arena (the 1990s popular uprisings and fall of Presidents, noted above) and indigenous candidates were successful in local and national elections. As various respondents noted, the political clout achieved by the indigenous movement enabled them to put forward their own candidates for key positions such as hospital directorates. Respondents underlined how the presence of indigenous actors within Otavalo’s maternal health policy community was vital in explaining the emergence of the VB practice:

If we didn’t have an indigenous Mayor, if these [indigenous professionals] were not in these different positions, we wouldn’t have the results we have today (…) because we opened up spaces … Twenty years ago it was very difficult to access the hospital because of the racism and discrimination… but [things changed] because there were indigenous health professionals who led the [intercultural health] project … and then there are political changes, it was unimaginable [before] to have an indigenous [hospital] director, so opening up those spaces helped to change things. (Manager 1, indigenous)

In addition, research findings show that many of these indigenous professionals working at the local level had links with the national as well as local indigenous movement and expressed their activism through their jobs. For instance, an indigenous respondent reported how his own experience of ethnic discrimination in his youth influenced his career choice. He eventually got involved in the indigenous movement and took up a position as a health manager in a local health unit where he felt he could advance the goals of the indigenous movement for greater equity. Like him, other indigenous respondents noted that gaining space within official health institutions was a way of meeting the indigenous movement’s goals by affecting real change. As another indigenous respondent reported:

When I finished my degree I got involved with the indigenous movement; I got to know the political part and the needs of the [indigenous] community (…). As part of the indigenous movement, we thought that we should take positions that were, in inverted commas, “forbidden” for indigenous peoples; positions where we could have decision-making power, as any other citizen. If we want to change health services, to change people and recognition for traditional medicine, we need to get in there and fight. We should not isolate ourselves … (Manager 2, indigenous)

### Defining maternal mortality as a problem of ethnic inequity

Our results show that this influential maternal health policy community was formed by large numbers of indigenous people; they were instrumental in identifying MMR amongst indigenous women in the region as a public health problem arising from ethnic inequities, defining its causes and the subsequent events that led up to the VB practice .

Several respondents explained that the problem of MMR in Otavalo came to the fore in 2003 as maternal mortality registration improved in the region. Various indigenous respondents recalled how alarmed they were by the scale of the problem, so they conducted further investigations in rural areas which showed severe ethnic inequities in MMR. It was found that geographical and financial barriers, poor functioning of the health system, and women’s delays in recognising and acting upon obstetric complications were important contributors to MMR but did not fully explain indigenous women’s low levels of access to services. For respondents within the maternal health policy community, the key issue was that widespread discrimination against indigenous women within the health service prevented them from accessing services. Indigenous respondents were more likely to frame indigenous MMR within a larger discourse of indigenous rights than mestizo respondents. As they explained, ethnic discrimination was rooted in Ecuador’s history; since colonial times, indigenous people lived in a situation of institutionalised social exclusion and disadvantage. Indigenous respondents highlighted the important role played by the indigenous movement that since the 1990s had fought to demand better living conditions for the indigenous community.

[Healthcare workers] said that the vertical birth damaged women’s health … Yet, we say that what [healthcare workers] do in hospitals is bad because they maltreat babies and women and they don’t provide adequate care. Now, the Ministry of Health has recognised [traditional medicine] and for [the indigenous movement] it is very important because it shows that we are making advances in achieving our right to health. (Indigenous leader)

Several indigenous respondents reported that the indigenous movement first focused its demands on their right to access land and education. They described how later, buoyed on by their success to influence these policy areas, the indigenous movement began to rally against ethnic discrimination in other areas such as the health service.

*In the community workshops we said that we, as citizens, have the same right to access [healthcare], and if [health workers] don’t see us, we have to demand it. Because before, that was the worst part, if you made demands [mestizo people] said that this was because you were an Indian*^2^* troublemaker. (Manager 3, Indigenous)*

Only one respondent within the maternal health policy community had a different stand. This respondent was a mestizo manager working for the MOH who stated that his organization had been working for a long time in intercultural health and had prepared the ground for the VB practice. This respondent stated his support to intercultural health practices such as the VB to reduce MMR and to meet the MDGs. However, he reported that there was no discrimination in the health service and that indigenous peoples’ low access to health services should be attributed to factors such as indigenous preference for traditional medicine and geographical and language barriers.

We conduct the same health campaigns for mestizo and indigenous people, the problem is that then [indigenous people] don’t access health units. [Indigenous people] are told about interculturality but they are the most inflexible… it’s very difficult … all mestizo health professionals are open to see anyone; there is no discrimination (…). It is not the case that [mestizo health professionals] see mestizo people first but they see first whoever comes first so there isn’t any kind of discrimination. (Manager, mestizo 4)

In spite of this discrepancy, it was the view of the majority of those within the maternal health policy community that prevailed. They not only identified and raised awareness about indigenous maternal mortality in the region but were also successful in framing it as an issue of ethnic inequity and indigenous rights. This was critical for the eventual emergence of the VB practice as a culturally appropriate solution.

### Policy stream: identifying the vertical birth as a solution

The maternal health policy community reported that the formulation of the VB practice as a ‘solution’ to indigenous health inequities benefitted from being able to build on the implementation of previous initiatives to address MMR over several years. Respondents highlighted the following: the 1998 Constitution, the Free Maternity Law (FML), a ‘maternal survival’ pilot initiative implemented by CARE International, and the Jambi Wasi intercultural health initiative. Interview data show that the implementation of these initiatives was important as it promoted collaboration between different actors in the maternal health policy community working in different institutions with responsibility for maternal health including the local government, the MOH and other institutions. Through the implementation of these initiatives in Otavalo, the maternal health policy community gained experience in maternal health and consolidated links with powerful actors and institutions that made important contributions to the eventual emergence of the VB practice. For example, local actors worked closely with the UNFPA which had funded the Jambi Wasi for many years. The UNFPA also provided expertise and resources to adapt the hospital infrastructure and to conduct an initial needs assessment in maternity services that was used to formulate the VB practice.

### 1998 constitution

The VB practice emerged shortly before the current Constitution was passed in 2008 which places a stronger emphasis on interculturality and traditional medicine. Nonetheless, the 1998 Constitution already defined Ecuador as a multicultural country and recognised the need to respect and promote traditional medicine which was used to support and justify the VB practice, according to respondents.

#### The free maternity law (FML)

Passed in 1994, this was a national policy to provide free reproductive and sexual healthcare for women and care for children under-5 in a context of cost-recovery in the health service. According to respondents, research findings into maternal deaths were used to galvanise support and demand action from the local and national institutions that had responsibilities under the FML. Despite many implementation challenges, respondents said the FML had provided a legal framework to start addressing MMR in Otavalo, an inter-institutional forum to discuss maternal issues and, eventually, part of the funding to implement the VB practice.

In 2003 the number of maternal deaths became evident because registration systems improved. It was like awakening to the maternal deaths issue and further research was conducted (…). This was the starting point to demand greater commitment from the [local] hospital to avoid maternal deaths (…). We had meetings to set up the Free Maternity Law management committee; also the current Mayor was leading the local government and CARE [International] put forward a proposal for the maternal survival pilot project. Through the [Free Maternity Law] management committee we continued to work on some of the activities started with the CARE [International] project to reduce maternal mortality. (Policy-maker 1, indigenous)

#### CARE international’s maternal survival pilot project

This was a small pilot project in five communities where there were maternal deaths. Respondents reported that the MOH and local government collaborated to identify TBAs working in these communities, provide them with training and basic equipment and strengthen the links between the MOH and the TBAs. TBAs were to become a core component of the VB practice.

#### Intercultural health in jambi wasi

Another project that respondents cited was the intercultural health project in Jambi Wasi in Otavalo. The Jambi Wasi was a non-for-profit clinic belonging to the indigenous movement that offered both traditional and biomedical medicine, including reproductive health services and had close links with the UNFPA. The Jambi Wasi implemented a precursor of the VB practice, the *‘humanized birth’*, which respondents said had emerged as a response to indigenous women’s demands for better care during labour. Women were allowed to give birth according to their tradition (e.g. mobilising during labour, delivering upright and having a birth companion and a TBA) and were treated with respect. For respondents, the *‘humanized birth’* experience in Jambi Wasi had been very positive but the lack of resources at that clinic limited scale-up and they had concluded that the best solution was to implement indigenous birthing practices in the local hospital.

The first idea was to implement the vertical birth in the Jambi Wasi but it was not possible [for the Jambi Wasi] to take on that responsibility; it was a big responsibility because of budgetary, staff and infrastructure implications. […] we saw that the [vertical birth] was needed because women attending the [Jambi Wasi] for antenatal care, mainly with the traditional birth attendants, asked to deliver there but it wasn’t possible; that is why it was suggested that the [vertical birth] should be implemented in the hospital. (Manager 5, mestizo).

According to our document review, the VB practice was a local interpretation of a broader national policy to reduce maternal and neonatal mortality, although this and other relevant laws and policies (Gobierno Ecuador 2006, 2007; MSP 2008b) were not brought up by respondents. Nevertheless, these initiatives provided an opportunity for the maternal health policy community to reach common understanding of the need for culturally sensitive responses to reduce ethnic maternal health inequities. This gave rise to a critical mass of supportive actors ready to respond to political triggers and seize a window of opportunity that allowed the VB practice to emerge.

### Politics stream: change of national government champions intercultural health

For many respondents the most dramatic policy development for intercultural maternal health in Otavalo was triggered by the change of government in 2007. That year Rafael Correa won the national elections with significant backing from indigenous voters (Bowen 2011). Soon after the election a new constitution was passed. The new constitution contained some of the demands of the indigenous movement encapsulated in the principle of *‘Sumak Kawsay’* or *‘Good Living’*, an indigenous principle that promotes equitable development and balance between humans and nature (Maldonado Ruiz 2010). As respondents reported (though some questioned the political motivations), in the health arena the indigenous principle of *‘Sumak Kawsay’* was translated into a greater emphasis from the State in intercultural health policies:

This government had as a strategy to conquer everybody’s hearts. One of their alliances was with the indigenous movement and [Correa’s party] looked for things to introduce in its discourse that could help [to win] votes. Along the way some [policies] have worked and others have been diluted. This ‘Good living’ has been well integrated by the Ministry of Health so the intercultural health [department] has had the chance to put issues on the agenda and now they are very involved with the cultural adaptation of health services. (Policy-maker 2, mestizo)

At the same time, respondents reported that the MOH had given greater attention to decreasing the MMR, partly as equity and global policies such as the MDGs started to gain relevance in the domestic agenda:

One of the reasons why there is so much emphasis on intercultural [health] issues is because, if you don’t make health services responsive, respecting cultural patterns (…) the gap between [the population and the health service] will always be too big. (…) We also work towards achieving the millennium development goals, namely child nutrition, poverty and maternal mortality which need to be reduced (…). For the ministry of health, the maternal health component is very important. (Policy-maker 3, mestizo)

Respondents reported that the new government had brought about positive changes to the MOH such as greater resources, a new strategic vision, and changes to the MOH leadership at national, provincial and local level. At the local level, respondents pointed out that the single most important factor to forward intercultural health in the public health system was the appointment, for the first time, of an indigenous doctor as Otavalo’s hospital director. He was part of the maternal health policy community and had the support of other members of the maternal health policy community, the indigenous movement and the MOH leadership.

With the change of government the maternal health policy community identified and seized a political window of opportunity to propose the implementation of VB in Otavalo’s public hospital. As a respondent summarised it:

What is important are the previous experiences [in intercultural health], the organization capability in Otavalo, a strong political will from the authorities, a strong social fabric…. those factors are very important. And the political will…. when we started to work in the hospital there was, for the first time, an indigenous director… thus, there were a set of conditions telling us that this was the time and the place to implement the ‘vertical birth’. (Policy-maker 4, mestizo)

Otavalo became one of the most visible examples of implementing intercultural health in the country and a key actor of the Otavalan maternal health policy community took up a leading role in the intercultural health department of the national MOH. The hospital was visited by numerous national and international delegations wanting to see first-hand how the VB practice had been implemented and adapt it to their context. Thus, it seems to be an iterative process at work: the emergence of the VB practice was indeed facilitated by developments in intercultural health policy at the national level. At the same time, Otavalo’s local experience appears to have had great influence over the development of a national intercultural maternal policy.

Whilst respondents acknowledged the importance of recent political events to catalyse policy change, some pointed out that change would only be sustained if policies addressing ethnic inequities were grounded in rights and those rights were internalised by society at large.

There is a need to ensure that things don’t work only because someone is there and if that person goes the whole thing is over. We need to realise that there are two cultures living together here; that is the reality. We all need to change our attitude, we need to internalise this. (Manager 1, indigenous)

## Discussion and conclusions

Overall, our study found that Kingdon’s model provided useful insights when analysing the emergence of the VB practice (policy with a small p) in Otavalo. Consistent with Kingdon’s model our results indicate that policy change resulted from the convergence of the problem, policy and politics streams in a window of opportunity that policy entrepreneurs were able to take advantage of. “Punctuated equilibrium” is an important concept that deepens Kingdon’s original theory in understanding the policy processes. According to this, the policy process is marked by prolonged periods of stability followed by sudden and profound shifts that lead to policy change (Baumgartner and Jones 1993). Kingdon points out that both gradualistic and punctuated equilibrium seem to be at work in different parts of the process (Kingdon 2003). Consistently, our results show that the processes of defining the policy problem and developing alternatives were gradual but that political changes (i.e. change of national government) catalysed the emergence of the VB practice. How change may happen in each stream is an important issue that deserves greater attention in future research. Our results confirm the importance of statistics in identifying potential policy problems (Kingdon 2003; Jat *et al.* 2013). As registration of maternal deaths improved in the region, actors became alarmed by the scale of the problem and demanded action from institutions responsible for maternal health services (e.g. hospital, local government).

The various maternal health policies and initiatives that followed showcased possible policy alternatives and prepared the foundation for the eventual emergence of the VB practice in Otavalo. The inter-institutional coordination required to implement these initiatives gave rise to a maternal health policy community. This maternal health policy community was partially comprised of indigenous health professionals who had been able to access education due to socio-economic and political changes in the region. Over the years, this policy community developed a significant expertise in maternal health. Their involvement in initiatives allowed them to identify what policy elements were most valuable, gaps that needed to be addressed, and to propose a feasible policy solution when the opportunity arose. For instance, some of the components of the VB practice such as the articulation of TBAs, and traditional and western medicine had already been implemented before with considerable success in ‘humanized birth’ in Jambi Wasi.

Sustained activity around maternal health contributed to keep this issue on the local agenda and ensured the presence of ‘policy entrepreneurs’ who identified a window of opportunity and pushed for policy change when the time was ripe. This opportunity came with the change of government as politicians were eager to back a practice that encapsulated their own values and political goals; that is, equitable access to health services and reduction of MMR. However Kingdon’s model, which was developed in the United States, has some limitations when applied to other settings. First, it fails to take into account different ways in which actors other than Kingdon’s “policy elites” may influence the policy process in other political systems—a point that has also been made by others (Kenney 2003; Jat *et al.* 2013; Balarajan 2014). Second, it does not adequately recognise the importance of the local socioeconomic and political historical contexts which shape not only the actors that emerge as policy actors and entrepreneurs but also the interpretation of the policy problem and solution.

Our case study indicates that the presence of a strong social movement, in Otavalo the indigenous movement, can be crucial actor in agenda setting, as studies in other countries have found (Kenney 2003; Brown *et al.* 2004; Jat *et al.* 2013) but was not recognised in Kingdon’s original model. Furthermore, our study additionally highlights the importance of historical context in the shaping of non-elite actors who later become critical in policy agenda setting. At the national level, the indigenous movement had become a key political influence in Ecuador after more than twenty years of organizing efforts for greater political representation and not least because of their involvement in the toppling of national governments during the 1990s. As a result, political elites were forced to make some concessions to the indigenous movement (Lalander and Gustafsson 2008; Bowen 2011). Certainly, Rafael Correa sought to secure the indigenous support in his first presidential election bid and introduced some of their demands in his political discourse. Our respondents noted that in return for the indigenous support, an indigenous doctor was appointed as a hospital director which was a key factor in the emergence of the VB practice.

The influence of the indigenous movement at the local level was also important. The maternal health policy community was comprised of indigenous actors who were involved in the indigenous movement or shared their demands for greater ethnic equity. These indigenous actors tried to advance the goals of the indigenous movement and expressed their activism through their jobs as managers, policy-makers or community leaders. They not only identified MMR amongst indigenous women as a problem; they were also critical in framing it in a wider discourse of ethnic discrimination and indigenous rights.

The ability of local actors within the maternal health policy community to strategically partner with the UNFPA also facilitated the emergence of the VB practice in Otavalo. The UNFPA was not a government insider but did work closely with the MOH headquarters. The UNFPA had supported local initiatives to address MMR long before the VB practice (e.g. the Jambi Wasi) and acted as an intermediary between local actors and the MOH headquarters. In this way, local actors were able to put forward their preferred policy solution (VB). Though beyond the scope of this study, our findings and document review clearly show that the VB practice in Otavalo influenced the subsequent development of national policy on intercultural maternal health. For example, an influential actor in the emergence of the VB in Otavalo took up a leading role in the intercultural health department of the national MOH. Further, the importance of the VB practice in Otavalo is acknowledged in the national guidelines for culturally appropriate maternity care (MSP 2008a) and it was also evident by the interest of national and international delegations that visited the hospital and showed interest when it was presented at international conferences. Certainly, the VB practice seems to have gathered momentum and has already been replicated in other areas of Ecuador (MSP 2011; Ortiz 2013; Argoti 2014). Thus, agendas are not only set by political elites as Kingdon envisages; the VB practice emerged on the national stage as a core part of the national indigenous movement’s aims of greater intercultural respect and at a local level was driven by local champions, particularly strong in Otavalo, who interacted with key influential actors at different levels to pioneer the VB implementation as well as further shaping national policy and rollout.

Our study points towards another limitation of Kingdon’s model of agenda setting. Kingdon contends that policy entrepreneurs ‘lie in wait’ for windows of opportunity to appear in order to advance their policy proposals. Shiffman (2003) however argues that Kingdon’s model may have underestimated policy entrepreneurs’ ability to shape events. Consistently, our study indicates that indigenous actors changed the political landscape and opened up spaces to influence the political process. Again our study highlights the importance of historical as well as contemporary socio-economic and political contexts in shaping who these entrepreneurs are and what they are able to achieve. Otavalans’ position as successful global merchants created an optimum environment to achieve meaningful political participation at the local level. Indigenous people took positions in which they had decision-making power (e.g. Mayor, hospital director) and shifted, to some extent, power imbalances. This allowed indigenous actors to integrate their values into the VB practice and thus address underlying causes of ethnic inequities in the health service. Indeed, meaningful participation of affected communities has been found to affect policy and programs outcomes (Ross and Williams 2002; Tucker *et al.* 2013). In Mexico, e.g. researchers found that indigenous women did not use an intercultural birthing house because it had been planned and implemented without input from community leaders and stakeholders (Tucker *et al.* 2013). Participation should be a priority for those wishing to replicate similar intercultural health policies in other settings.

In spite of the palpable influence of the indigenous movement in Ecuador’s political life, Bowen (2011) warns that their gains are limited because political elites have been very skilful in diminishing the indigenous threat to the existing social hierarchy through incorporating them into a political and economic system that undermines indigenous efforts to address underlying inequities (Bowen 2011). Thus it seems Kingdon’s policy elites do indeed retain some ultimate powers which may temper the progress of policies to achieve greater equity.

## Notes

1. We use the term inequities in line with WHO definition to signify ‘differences in health status or in the distribution of health determinants between different population groups that are unnecessary and avoidable as well as unjust and unfair’ http://www.who.int/hia/about/glos/en/index1.html

2. In Ecuador, the word ‘Indian’ is used as a pejorative term for indigenous people.
